# Virtual monochromatic dual-energy CT reconstructions improve detection of cerebral infarct in patients with suspicion of stroke

**DOI:** 10.1007/s00234-020-02492-y

**Published:** 2020-07-29

**Authors:** Fasco van Ommen, Jan Willem Dankbaar, Guangming Zhu, Dylan N. Wolman, Jeremy J. Heit, Frans Kauw, Edwin Bennink, Hugo W. A. M. de Jong, Max Wintermark

**Affiliations:** 1 Department of Neuroradiology, Stanford University Medical Center, Palo Alto, CA USA; 2 Department of Radiology and Nuclear Medicine, University Medical Center Utrecht, E01.132, P.O. Box 85500, 3508 GA Utrecht, the Netherlands; 3 Image Sciences Institute, University Medical Center Utrecht, Utrecht, the Netherlands

**Keywords:** Stroke, Non-contrast CT, Dual-energy CT, Virtual monochromatic images

## Abstract

**Purpose:**

Early infarcts are hard to diagnose on non-contrast head CT. Dual-energy CT (DECT) may potentially increase infarct differentiation. The optimal DECT settings for differentiation were identified and evaluated.

**Methods:**

One hundred and twenty-five consecutive patients who presented with suspected acute ischemic stroke (AIS) and underwent non-contrast DECT and subsequent DWI were retrospectively identified. The DWI was used as reference standard. First, virtual monochromatic images (VMI) of 25 patients were reconstructed from 40 to 140 keV and scored by two readers for acute infarct. Sensitivity, specificity, positive, and negative predictive values for infarct detection were compared and a subset of VMI energies were selected. Next, for a separate larger cohort of 100 suspected AIS patients, conventional non-contrast CT (NCT) and selected VMI were scored by two readers for the presence and location of infarct. The same statistics for infarct detection were calculated. Infarct location match was compared per vascular territory. Subgroup analyses were dichotomized by time from last-seen-well to CT imaging.

**Results:**

A total of 80–90 keV VMI were marginally more sensitive (36.3–37.3%) than NCT (32.4%; *p* > 0.680), with marginally higher specificity (92.2–94.4 vs 91.1%; *p* > 0.509) for infarct detection. Location match was superior for VMI compared with NCT (28.7–27.4 vs 19.5%; *p* < 0.010). Within 4.5 h from last-seen-well, 80 keV VMI more accurately detected infarct (58.0 vs 54.0%) and localized infarcts (27.1 vs 11.9%; *p* = 0.004) than NCT, whereas after 4.5 h, 90 keV VMI was more accurate (69.3 vs 66.3%).

**Conclusion:**

Non-contrast 80–90 keV VMI best differentiates normal from infarcted brain parenchyma.

**Electronic supplementary material:**

The online version of this article (10.1007/s00234-020-02492-y) contains supplementary material, which is available to authorized users.

## Introduction

Non-contrast CT is the mainstay in the initial evaluation of patients with suspicion of acute ischemic stroke (AIS) [1]. However, the sensitivity of non-contrast CT is limited for the detection of acute brain infarct [2]. The reference standard for infarct detection is MRI with diffusion-weighted imaging (DWI) [3, 4]. DWI allows for detection of cytotoxic edema within infarcted tissue with high sensitivity, while CT is limited to detecting subtle changes in water content between infarcted and normal brain parenchyma.

Dual-energy CT (DECT) advances CT imaging by improving upon conventional non-contrast head CT (NCT) by acquiring data with two separate energy spectra, which allows improved contrast resolution, reduced image noise and beam-hardening artifacts, and spectral separation of constituent materials at equivalent dose [5, 6]. Virtual monochromatic CT images (VMI) can be derived from source DECT data [7–9], and reflect the tissue properties of a scan acquired at a single specific monochromatic energy level. Leveraging different monoenergetic reconstructions may help accentuate differences in energy-dependent attenuation differences between similar materials, e.g., normal brain tissue and ischemic/edematous brain tissue. DECT VMI have demonstrated improved contrast-to-noise profiles and reduced beam-hardening artifact relative to NCT [10–15]. The potential of DECT for the visualization of brain edema has been investigated in earlier studies [16–18]. In these studies, elaborate reconstruction methods were required, whereas VMI is a standard derivative of DECT imaging and broadly applicable to any vendor’s software. The use of VMI in the detection of cerebral infarct has not been established. We sought to identify the non-contrast VMI energy which best differentiates normal from infarcted brain parenchyma, and to determine if this VMI can more sensitively and specifically identify infarct in patients with suspected AIS compared with NCT.

## Materials and methods

### Patients

This study was approved by the Stanford University Institutional Review Board which waived the need for informed consent, and data collection complied with the Health Insurance Portability and Accountability Act. We retrospectively enrolled consecutive patients between October 13, 2018, and April 18, 2019, with suspected AIS who underwent non-contrast DECT and subsequent MRI with DWI within 48 h. Inclusion criteria were as follows: patient age > 18 years and presentation within 24 h of symptom onset. Exclusion criteria were as follows: technical failure of DECT, significant metal artifact limiting interpretation, or corrupted DWI. Baseline clinical data was collected, including age, sex, presentation National Institutes of Health Stroke Scale (NIHSS), time since last known well, time to initial DECT imaging, and time to subsequent MRI.

### Imaging protocol

All patients were imaged using a dual-source Somatom Flash CT scanner (Siemens Healthineers, Erlangen, Germany). Non-contrast dual-energy CT protocols are dose neutral with respect to single-energy acquisitions at our institution. The volume CT dose index (CTDIvol) for each scan was 59.8 mGy, and was based on a 16-cm International Electrochemical Commission (IEC) head dosimetry phantom. The scan parameters were as follows: Tube A, 80 kVp and 640 mAs; Tube B, 140 kVp with a tin filter and 320 mAs, beam collimation of 40 × 0.6 mm, a 1.0-s rotation time, matrix size 512 × 512, and a pitch of 1.0. Images were reconstructed at 3 mm using a medium smoothing Q34s kernel. Non-contrast DECT VMI images were reconstructed using the Monoenergetic+ software module in Syngo Via (Siemens Healthineers, Erlangen, Germany).

### Study design

Reference standard MRI (DWI) was obtained within 48 h of each patient’s index CT and was reviewed by a single neurointerventional radiologist with 7 years of experience (JJH). DWI imaging parameters included the following: TR 6000 ms, TE 78.2 ms, b-values 0 and 1000, flip angle 90°, and 5-mm slice thickness. Each MRI was scored on a binary scale for presence of cerebral infarct, defined as focal parenchymal restricted diffusion. Cerebral infarcts were binned by location using a modified Alberta Stroke Program Early CT Score (mASPECTS) [19, 20], in which additional regions corresponding to the posterior circulation (thalamus, superficial PCA, brainstem, and cerebellum) and anterior cerebral artery territory (A1 anteriorly, and A2 posteriorly) are added. The A1 territory is anatomically bounded as the inferior ACA territory, while the A2 territory was bounded by the superior ACA territory.

Phase 1 of this study was used as an initial selection step to identify the VMI reconstruction energies best suited for infarct detection. Phase 2 compared these identified VMI reconstructions against NCT for the detection of cerebral infarct and qualitative infarct localization to identify VMI energies best for differentiation between normal and infarcted brain tissue.

#### Phase 1 design

In the first phase of the study, VMI were reconstructed at 10 keV increments from 40 to 140 keV. VMI reconstructions were randomized and reviewed by a neuroradiologist and a neurologist (JWD and GZ, with 12 and 19 years of experience in radiology, respectively, and both more than 10 years of experience in stroke imaging) who were blinded to the reconstruction type, but did have CT indication information. Each reader reviewed a full axial image stack for each series with fixed window level and width settings. As attenuation values (HU) vary with tube voltage [21], the fixed window level (WL) for each reconstruction energy was visually identified by a neuroradiologist (MW) and set to maintain similar attenuation uniformity between series. The settings were as follows: 40–50 keV (WL 50), 60–70 keV (WL 45), 80–110 keV and NCT (WL 40), and 120–140 keV (WL 35). Window width (WW) was set to 50 HU for all reconstructions. Reviewers evaluated for the presence of acute cerebral infarct, and localized infarct foci using the mASPECTS system. Acute infarct was defined as a focal loss of gray-white matter differentiation, focal edema, or hypoattenuation without volume loss. Discrepant reviews were resolved by consensus. A number of VMI reconstructions, who were the most sensitive and specific for the detection of cerebral infarct relative to the reference standard MRI with DWI, were selected and used for comparison with NCT and used to identify the VMI energies that best differentiated normal from infarcted tissue.

#### Phase 2 design

In the second phase, two different independent reviewers scored a separate, larger cohort of patients presenting with suspicion for AIS. Each examination was reconstructed as a NCT (WL/WW 40/50) and the most sensitive and specific VMI energies identified in phase 1 (60–90 keV), and were presented in a randomized, blinded fashion using the same fixed window and level settings as described in phase 1. An experienced neuroradiologist (JWD) and a neuroradiology fellow (DNW) scored each examination for evidence of acute infarct. Subgroup analyses of patients imaged in the early window (≤ 4.5 h after last-seen-well) and those presenting in the late window (> 4.5 h after last-seen-well) were performed to assess for time-dependent differences in infarct detection. We expect that infarcts in the early window require different improvement in differentiation between similar attenuating tissue (fat, water, and soft tissue), then infarcts in the late window, and might have an influence on the required VMI energy for infarct detection. The volume of cerebral edema increases over time resulting in increased hypoattenuation on CT. At early time points, the contrast between the edema and normal brain tissue is therefore less conspicuous than at later time points. Increasing the contrast between edema and normal brain tissue at different time points may therefore require different energies.

### Statistical analysis

Baseline patient characteristics were compared between phase 1 and 2 using a Mann-Whitney signed rank test for continuous variables and a chi-squared test for discrete variables. In phase 1, the sensitivity, specificity, positive predictive value (PPV), and negative predictive value (NPV) were calculated for the detection of cerebral infarct for each scored VMI reconstruction relative to the reference standard MRI with DWI. In phase 2, the sensitivity, specificity, PPV, NPV, and accuracy for the detection of cerebral infarct were calculated for the selected VMI reconstruction and the NCT relative to the reference DWI. Sensitivity and specificity were compared between NCT and VMI using the chi-squared test. Regional infarct localization between DWI and CT was calculated as the infarct location match (ILM), which is defined as the percentage of true positive detections of infarct on CT compared with the total of positive detections for infarct on DWI in an anatomical region. ILM was compared between NCT and VMI for the anterior cerebral artery (ACA; A1 and A2), basal ganglia and insular cortex (BG; caudate, lentiform nucleus, internal capsule and insular cortex), posterior circulation (PCA; thalamus, superficial PCA, cerebellum and brainstem), middle cerebral artery at the level of the basal ganglia (sub-MCA; M1, M2 and M3), middle cerebral artery at the level of the ventricles immediately above the basal ganglia (sup-MCA; M4, M5 and M6), and total of all regions combined using McNemar’s test. Statistical significance was set at *α* = 0.05. To correct for multiple comparisons, a Bonferroni correction was applied. Inter-rater reliability was evaluated using Cohen’s Kappa, for which the following interpretations were used: slight (0.01–0.20), fair (0.21–0.40), moderate (0.41–0.60), good (0.61–0.80), near perfect (0.81–0.99), and perfect agreement (1). Subgroup analyses of early and the late window patients were performed using the same methods. Statistical analyses were performed in SPSS (version 25.0, IBM, New York).

## Results

### Patient characteristics

One hundred and twenty-five consecutive patients suspected of AIS were included (median age 65.0; IQR 51.8–80.3 years; 48% female). In phase 1, 25 patients were included (median age 67.0; IQR 54.5–75.3 years; 56% female), while in phase 2, a total of 96 patients (median age 63; IQR 52.5–81.0 years; 46.9% female) were included after exclusion of 4 patients for metallic artifact (1 CT case) and corrupted DWI images (3 MRI cases). Phase 1 patients had a median presenting NIHSS of 4 (IQR 1–11), while phase 2 patients had a median presentation NIHSS of 5 (IQR 2–12). Baseline patient demographics are summarized in Table 1. There were no significant differences in baseline patient characteristics between the groups.

**Table 1:** Patient characteristics of patients in phase 1 and phase 2 of the study

Characteristics	Phase 1 (*n* = 25)	Phase 2 (*n* = 96)	*P* value
General			
Sex, male:female	11:14	51:45	0.416
Age (years), median (IQR)	67.0 (54.5–75.3)	63.0 (52.5–81.0)	1.000
Presentation NIHSS, median (IQR)	4 (1–11)	5 (2–13)	0.700
Platelets, median (IQR)	201 (167–261)	222 (185–275)	0.700
Cerebral infarct on DWI, *n* (%)	12 (48.0)	51 (53.1)	0.648
Time since last-seen-well (hours), median (IQR)	5.0 (1.4–12.0)	4.2 (1.9–12.0)	1.000
Time CT to MR (hours), median (IQR)	5.0 (3.1–11.0)	5.2 (3.2–12.7)	0.700
IV tPA administered, *n* (%)	4 (16.0)	12 (12.5)	0.222
Stroke risk factors			
Coronary artery disease (CAD), *n* (%)	4 (16.0)	13 (13.5)	0.753
Atrium fibrillation, *n* (%)	3 (12.0)	12 (12.5)	0.946
Diabetes, *n* (%)	7 (28.0)	23 (24.0)	0.677
Hypertension, *n* (%)	15 (60.0)	55 (57.3)	0.807
Hyperlipemia, *n* (%)	12 (48.0)	38 (39.6)	0.447
Prior cerebrovascular incident, *n* (%)	3 (12.0)	28 (29.2)	0.080
Prior intracranial hemorrhage, *n* (%)	1 (4.0)	10 (10.4)	0.320
Smoker, *n* (%)	3 (12.0)	25 (26.0)	0.138
Antiplatelet or anticoagulant, *n* (%)	12 (48.0)	47 (49.0)	0.932

### Phase 1: initial selection VMI energies for infarct detection

**Fig. 1:**
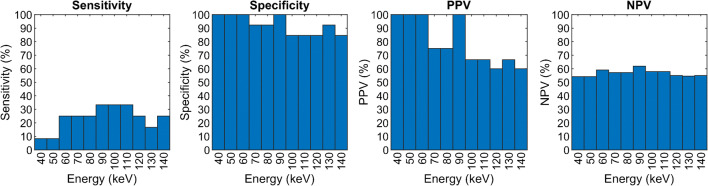
The sensitivity, specificity, positive predictive value, and negative predictive value of the consensus scoring of the different VMI reconstructions for detection of infarct

**Fig. 2:**
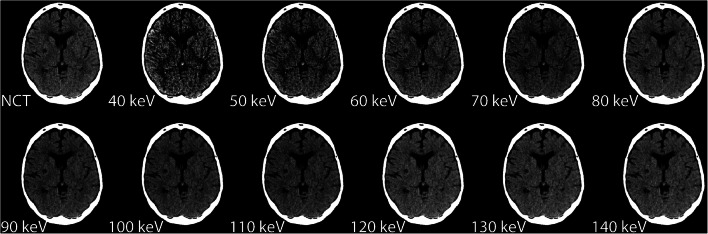
Example patient of VMI and conventional reconstructions. The NCT has a WL/WW of 40/50, 40, and 50 keV (WL/WW, 50/50), 60 and 70 keV (WL/WW, 45/50), 80, 90, 100, and 110 keV (WL/WW, 40/50), and 120, 130, and 140 keV (WL/WW, 35/50)

Acute cerebral infarct was identified in 12/25 (48%) patients by DWI. Sensitivity, specificity, PPV, and NPV of each VMI reconstruction from 40 to 140 keV for the detection of infarct relative to the reference DWI are shown in Fig. 1. The inter-observer variability range for the VMI reconstructions was 0.34–0.75. The 60–120 keV reconstructions showed the highest sensitivities (25.0–33.3%), but 60–90 keV also had a high specificity (92.3–100.0%), PPV (75.0–100.0%), and NPV (57.1–61.9%) with a moderate inter-reader agreement (0.41–0.45). Sensitivity was lowest for the 40, 50 and 130 keV VMI reconstructions (8.3-16.7%), whereas the 120 and 140 keV demonstrated lower specificity versus the 60-90 keV reconstructions (84.6% vs 92.3-100%). Figure 2 illustrates typical images the observers reviewed.

VMI reconstructions from 60 to 90 keV were the most sensitive and specific for the detection of cerebral infarct, and were chosen for further evaluation in phase 2.

### Phase 2: comparison of VMI with conventional CT

#### Infarct detection

**Fig. 3:**
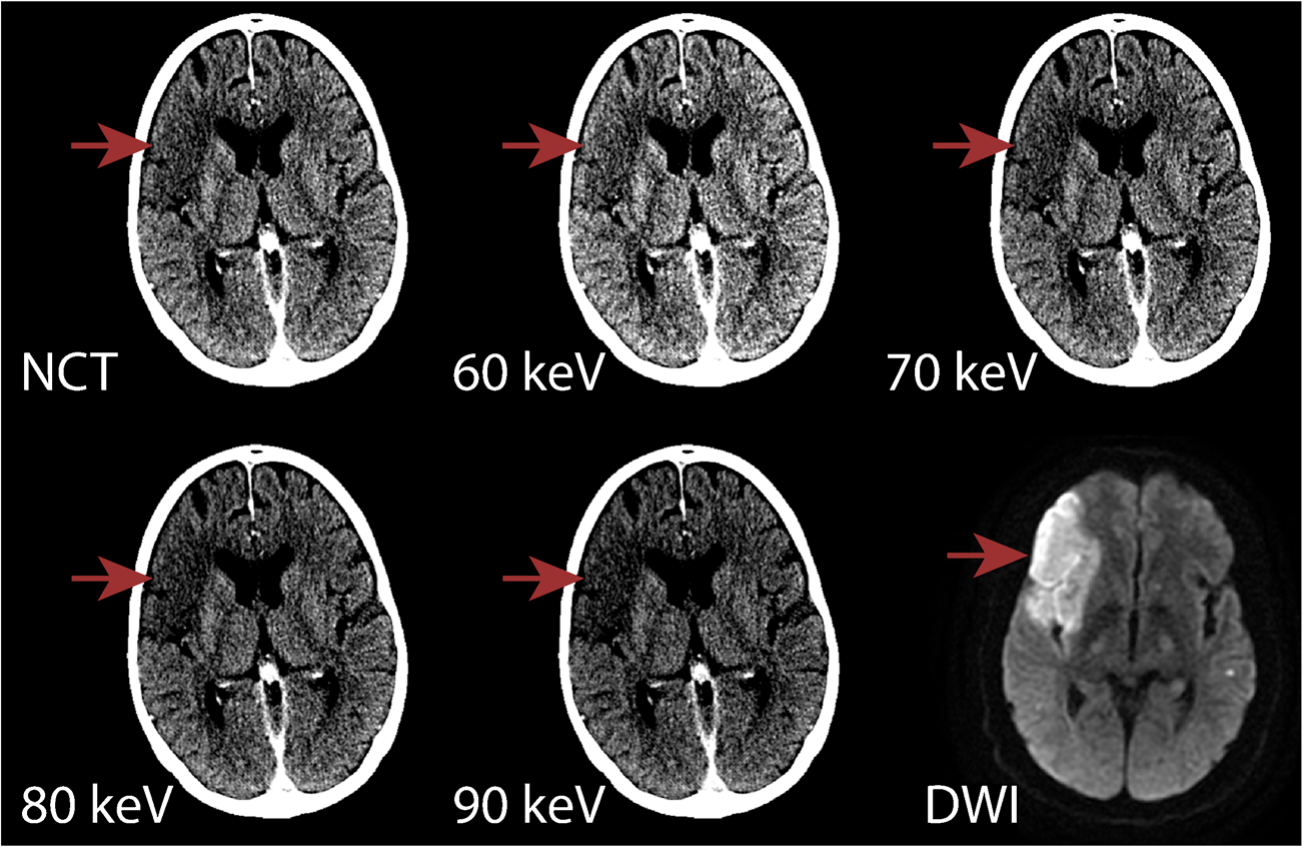
Example case of a patient demonstrating the increased conspicuity of an acute infarct on the 80 and 90 keV VMI in comparison to NCT, 60 and 70 keV and corresponding diffusion restriction on subsequent MRI DWI confirmed the territory of infarct. Red arrows indicate the outlining of the infarcted area. The CT reconstructions have the same WL/WW, 40/30

DWI identified 51/96 (53%) patients with acute infarction. The sensitivity, specificity, PPV, NPV, and accuracy for the detection of infarct for all included VMI reconstructions and the NCT are shown in Table 2. The VMI reconstructions (70–90 keV) had higher mean sensitivity relative to NCT, even though not significant (36.0–37.3 vs NCT 32.4%; *p* > 0.680). VMI reconstructed at 80 and 90 keV demonstrated higher sensitivity (36.0, 37.3 vs NCT 32.4%; *p* > 0.680), specificity (92.2, 94.4 vs NCT 91.1%; *p* > 0.509), PPV (84.0, 89.1 vs NCT 82.0%), NPV (55.7, 56.6 vs NCT 54.2%), and accuracy (62.0, 63.5 vs NCT 59.9%) compared with the NCT, where 90 keV performed best. Inter-rater reliability for all reconstructions was good (0.64–0.78). Figure 3 illustrates the difference in conspicuity of infarct between VMI and NCT in comparison with DWI.

**Table 2:** Infarct detection

	Sensitivity (R1, R2)	Specificity (R1, R2)	PPV (R1, R2)	NPV (R1, R2)	Accuracy (R1, R2)	IRR (95% CI)
NCT	32.4 (33.3, 31.4)	91.1 (97.8, 84.4)	82.0 (94.4, 69.6)	54.2 (56.4, 52.1)	59.9 (63.5, 56.3)	0.64 (0.51–0.77)
60 keV	30.4 (25.5, 35.3)	85.6 (95.6, 75.6)	74.4 (86.7, 62.1)	51.9 (53.1, 50.7)	56.3 (58.3, 54.2)	0.71 (0.60–0.83)
70 keV	36.0 (33.3, 38.7)	86.7 (93.3, 80.0)	77.0 (85.0, 69.0)	54.5 (55.3, 53.7)	59.9 (61.5, 58.3)	0.65 (0.52–0.78)
80 keV	36.0 (38.7, 33.3)	92.2 (97.8, 86.7)	84.0 (95.2, 72.7)	55.7 (58.7, 52.7)	62.0 (66.7, 57.3)	0.73 (0.61–0.85)
90 keV	37.3 (37.3, 37.3)	94.4 (100.0, 88.9)	89.1 (100.0, 78.3)	56.6 (58.4, 54.8)	63.5 (66.7, 60.4)	0.78 (0.67–0.89)

Infarct detection of VMI and conventional CT (NCT) shown in sensitivity (%), specificity (%), PPV (%), NPV (%), accuracy (%), and inter-reader reliability (IRR), with 95% confidence interval (CI). The sensitivity, specificity, PPV, NPV, and accuracy of each individual observer (R1, R2) are provided parenthetically after the pooled value

#### Infarct per territory

Pooled ILM of NCT and VMI are shown in Table 3. NCT and VMI were unable to match the location of infarct in the anterior cerebral artery to DWI. ILM-NCT was higher in the posterior circulation (11.1–13.3 vs 15.5%), though not significant. Other regions (sub-MCA, sup-MCA, and basal ganglia) were higher for 80 and 90 keV VMI compared to NCT, and these differences were significant in the middle cerebral artery at the level of the basal ganglia (sub-MCA; 29.6-33.3 vs NCT 7.4%; *p* < 0.015). ILM for 80 and 90 keV reconstructions for all regions was significantly higher compared to NCT (28.7 and 27.4% vs. 19.5%; *p* < 0.010). The inter-reader reliability was near perfect for all CT reconstructions (0.86–0.88).

**Table 3:** ILM comparison for anterior cerebral artery, middle cerebral artery, basal ganglia, and posterior circulation

Region	DWI (*n*)	ILM (%) (95% CI)				
		NCT	60 keV	70 keV	80 keV	90 keV
ACA	10	0.0 (0.0–12.5)	0.0 (0.0–12.5)	0.0 (0.0–12.5)	0.0 (0.0–12.5)	0.0 (0.0–12.5)
Sub-MCA	27	7.4 (2.1–23.4)	3.7 (0.7–18.3)	18.5 (8.2–36.7)	33.3 (18.6–52.2)*	29.6 (15.9–48.5) *
Sup-MCA	49	32.7 (21.2–46.6)	22.4 (13.0–35.9)	36.7 (24.7–50.7)	38.8 (26.4–52.8)	38.8 (26.4–52.8)
BG	33	21.2 (10.7–37.8)	21.2 (10.7–37.8)	39.4 (24.7–56.3)	39.4 (24.7–56.3)	36.4 (22.2–53.4)
PCA	45	15.6 (7.8–28.8)	13.3 (6.3–26.2)	11.1 (4.8–23.5)	13.3 (6.3–26.2)	13.3 (6.3–26.2)
All	164	19.5 (14.2–26.2)	15.2 (10.5–21.5)	25.0 (19.0–32.2)	28.7 (22.3–36.0)*	27.4 (21.2–34.7)*
IRR (95% CI)		0.87 (0.86–0.89)	0.88 (0.86–0.90)	0.87 (0.85–0.89)	0.86 (0.84–0.88)	0.86 (0.84–0.88)

Pooled ILM of VMI and NCT with 95% confidence interval (CI) are presented and compared. If the difference between NCT and VMI is significant, it is highlighted with an asterisk. In addition, inter-reader reliability (IRR) with 95% confidence interval is shown for each CT reconstruction. Regions: *ACA*, anterior cerebral artery (A1 and A2); *BG*, basal ganglia (caudate, lentiform nucleus, internal capsule and insular cortex); *PCA*, posterior circulation (thalamus, superficial PCA, cerebellum and brainstem); *sub-MCA*, middle cerebral artery at level basal ganglia (M1, M2, and M3); and *sup-MCA*, middle cerebral artery at the level of the ventricles immediately above the basal ganglia (M4, M5, and M6)

#### Infarct in the early and late windows

Fifty patients were scanned within the early window (median time 1.9, IQR 0.8–3.0 h). Among these patients, 25 (50.0%) had DWI-positive acute cerebral infarct. Forty-six patients were scanned within the late window (median time 12.0, IQR 6.3–17.9 h). Among these patients, 26 (56.5%) had DWI-positive acute cerebral infarct.

For early window patients, VMI reconstructions at 80 keV showed the highest mean sensitivity (26.0%), whereas NCT had 18.0% sensitivity. The difference, however, is not significant (*p* = 0.499). In addition, 80 keV VMI showed similar or higher specificity (90.0 vs 90.0%; *p* = 1.000), PPV (73.8 vs 64.3%), NPV (54.8 vs 52.3%), and accuracy (58.0 vs 54.0%) relative to NCT. A good inter-rater reliability was seen for all reconstructions (0.66–0.79). Patients in the late window showed higher sensitivity for 90 keV compared with NCT (50.0 vs 46.2%; *p* = 0.786). Specificity was higher for 90 keV compared with NCT (95.0 vs 92.5%; *p* = 0.747). PPV (93.3 vs 90.6%), NPV (0.59.3 vs 56.9%), and accuracy (69.6 vs 66.3%) are also higher for 90 keV compared with NCT. NCT and VMI showed good inter-rater reliability (0.61–0.77). Results of other VMI in early and late window are shown in Table 1 of supplementary materials.

Pooled ILM of NCT and VMI in the early- and late-windows are shown in Table 4. In the early window, location matching on VMI at 80 keV was similar or better than NCT in all regions (ACA, 0.0% vs. 0.0%, sub-MCA; 36.4% vs. 0.0%, sup-MCA; 43.8% vs. 25.0%, BG; 36.4% vs. 18.2% and PCA; 5.9% vs. 5.9%). The increase, however, was not significant in any of the regions. ILM of all regions of 80 keV VMI (27.1%) was significantly higher (*p* = 0.012) compared to total ILM of NCT (11.9%). The inter-reader reliability was near perfect for all CT reconstructions (0.83-0.85). In the late window, VMI at 90 keV outperformed NCT for all territories (ACA, 0.0% vs. 0.0%, sub-MCA; 37.5% vs. 12.5%, sup-MCA; 39.4% vs. 36.4% and BG; 40.9% vs. 22.7%), except in the posterior circulation (PCA, 17.9% vs. 21.4%). The differences were not significant. The ILM of all regions of 90 keV VMI (31.4%) was significantly higher compared to NCTILM of all regions (23.8%; *p* = 0.031). The inter-reader reliability was near perfect for all CT reconstructions (0.88–0.89).

**Table 4:** ILM comparison for anterior cerebral artery, middle cerebral artery, basal ganglia, and posterior circulation as a function of time

Region	DWI (*n*)	ILM (%) (95% CI)				
		NCT	60 keV	70 keV	80 keV	90 keV
≤ 4.5 h						
ACA	4	0.0 (0.0–49.0)	0.0 (0.0–49.0)	0.0 (0.0–49.0)	0.0 (0.0–49.0)	0.0 (0.0–49.0)
Sub-MCA	11	0.0 (0.0–25.9)	0.0 (0.0–25.9)	27.3 (9.7–56.6)	36.4 (15.2–64.6)	18.2 (5.1–47.7)
Sup-MCA	16	25.0 (10.2–49.5)	12.5 (3.5–36.0)	37.5 (18.5–61.4)	43.8 (23.1–66.8)	37.5 (18.5–61.4)
BG	11	18.2 (5.1–47.7)	0.0 (0.0–25.9)	9.1 (1.6–37.7)	36.4 (15.2–64.6)	27.3 (9.7–56.6)
PCA	17	5.9 (1.1–27.0)	5.9 (1.1–27.0)	5.9 (1.1–27.0)	5.9 (1.1–27.0)	5.9 (1.1–27.0)
All	59	11.9 (5.9–22.5)	5.1 (1.7–13.9)	18.6 (10.7–30.4)	27.1 (17.4–39.6)*	20.3 (12.0–32.3)
IRR (95% CI)		0.85 (0.82–0.88)	0.85 (0.83–0.88)	0.85 (0.82–0.87)	0.83 (0.80–0.86)	0.83 (0.80–0.85)
> 4.5 h						
ACA	6	0.0 (0.0–39.0)	0.0 (0.0–39.0)	0.0 (0.0–39.0)	0.0 (0.0–39.0)	0.0 (0.0–39.0)
Sub-MCA	16	12.5 (3.5–36.0)	6.3 (1.1–28.3)	12.5 (3.5–36.0)	31.3 (14.2–55.6)	37.5 (18.5–61.4)
Sup-MCA	33	36.4 (22.2–53.4)	27.3 (15.1–44.2)	36.4 (22.2–53.4)	36.4 (22.2–53.4)	39.4 (24.7–56.3)
BG	22	22.7 (10.1–43.4)	31.8 (16.4–52.7)	54.5 (34.7–73.1)	40.9 (23.3–61.3)	40.9 (23.3–61.3)
PCA	28	21.4 (10.2–39.5)	17.9 (7.9–35.6)	14.3 (5.7–31.5)*	17.9 (7.9–35.6)	17.9 (7.9–35.6)
All	105	23.8 (16.7–32.8)	21.0 (14.3–29.7)	28.6 (20.8–37.9)	29.5 (21.6–38.8)	31.4 (23.3–40.8)*
IRR (95% CI)		0.89 (0.86–0.91)	0.89 (0.86–0.92)	0.88 (0.85–0.90)	0.88 (0.85–0.90)	0.88 (0.85–0.90)

Pooled ILM of VMI and NCT with 95% confidence interval (CI) are compared in the early- and late-windows. If the difference between NCT and VMI is significant, it is highlighted with an asterisk. In addition, inter-reader reliability (IRR) with 95% confidence interval is shown for each CT reconstruction. Regions: *ACA*, anterior cerebral artery (A1 and A2); *BG*, basal ganglia (caudate, lentiform nucleus, internal capsule and insular cortex); *PCA*, posterior circulation (thalamus, superficial PCA, cerebellum and brainstem); *sub-MCA*, middle cerebral artery at level basal ganglia (M1, M2, and M3), and *sup-MCA*, middle cerebral artery at the level of the ventricles immediately above the basal ganglia (M4, M5 and M6)

## Discussion

In this study, VMI reconstructions at 90 keV showed higher sensitivity (36.3%) and specificity (94.4%) for the detection of acute cerebral infarct in AIS patients than NCT (32.4% and 91.1%, respectively). This VMI energy is concordant with prior reports suggesting that optimal VMI for maximum contrast resolution is between 60 and 100 keV in adults depending on the DECT approach [10, 14, 22]. Pomerantz et al. [10] suggested 65–75 keV for overall parenchymal contrast resolution, with agreement by Neuhaus et al. [14] at 65 keV for gray-white differentiation, whereas Yoshida et al. [22] suggested that 99 keV offered superior evaluation of supratentorial acute infarct. Yoshida et al., however, only used confirmed infarct patients; we included AIS patients. By comparing our CT findings with DWI, we were able to identify the best VMI energies for differentiating between normal and infarcted tissue. Additionally, VMI reconstructions at 90 keV also had the highest NPV (56.6 vs 54.2%). As CT is often used as a screening modality in cases where there is concern for AIS, improvements in the sensitivity and NPV offered by VMI may lend confidence to the neuroradiologist in excluding cerebral infarct, particularly if MRI is contraindicated or unavailable.

Subgroup analyses of patients imaged ≤ 4.5 h and > 4.5 h from last-seen-well were performed as infarct conspicuity changes with increasing edema in the maturing lesion. Analysis of patients presenting ≤ 4.5 h from last known well demonstrated a small improvement in sensitivity (26.0 vs 18.0) and NPV (54.8 vs 52.3%), and similar specificity (90.0%) for the detection of acute cerebral infarct using VMI at 80 keV as compared with NCT. Given that patients presenting within this window are considered eligible for intravenous thrombolytic therapy, even small improvements in infarct detection may increase clinical and therapeutic certainty [23].

Infarct localization relative to reference standard DWI was assessed using ILM for each pre-defined cerebral regions. Within the middle cerebral artery territories (sub-MCA and sup-MCA), a non-significant trend towards superior VMI-ILM at 80–90 keV was observed relative to NCT-ILM. However, overall VMI-ILM showed a significant improvement with 80–90 keV in comparison with total NCT-ILM (28.7, 27.4 vs 19.5%), which suggests that VMI may significantly increase the accuracy of infarct localization. Given regional improvement in infarct detection of VMI seen at 80 keV in the early window within the anterior circulation, assessment of VMI in an anterior large-vessel occlusion population is warranted. To determine if infarct core may be better estimated with VMI than with NCT and could be used for early window treatment decisions [24], in our study, we see a clear trend towards a significant increase in ILM using VMI; however, our study was underpowered given the number of territories surveyed, and further testing in a larger cohort is warranted.

Relative to NCT, VMI reconstructions at 80–90 keV showed a significant improvement of acute infarct detection. This improvement is most likely due to the qualitatively demonstrated improved soft tissue contrast. This improved contrast therefore increases small inherent attenuation differences between gray matter, white matter, and edematous tissue compared with NCT. We hypothesize that the increase in contrast is due to increased differences between attenuation of gray and white matter and to the lower noise levels in 80 and 90 keV VMI [15, 22]. The range of best VMI energies is 80–90 keV, and we recommend individual institutions to test their own optima, but 90 keV generally has a slightly better sensitivity and specificity for infarct detection compared with 80 keV. Infarct location detection is slightly better with 80 keV. However, if dual-energy CT is unavailable, similar results are unlikely to be obtained by modifying single-energy CT protocols to an average energy of 90 keV, as this will involve an increase in peak tube voltage and a substantial decrease in exposure to maintain a similar dose level. This increase in kVp will result in a decrease in contrast, because there will be a large number of photons with an energy higher than 90 keV which will not attenuate in tissue.

Our study has several limitations. First, our retrospective study design may introduce bias. The small sample size may limit our statistical analysis, particularly for analysis of ILM. Second, our sensitivity analysis may be artificially increased as the imaging reviewers were aware that the testing population was enriched for patients with cerebral infarct. Third, a recognition bias can be assumed, resulting in an underestimation of the differences between the reconstruction types. We, however, blinded our reviewers to reconstruction type, CT reconstructions were presented in a random fashion, and the large amount of CT reconstructions in the comparison ensured that no single energy level was favored over the other. Lastly, we investigated the applicability of VMI using a single type of DECT acquisition (dual-source CT scanner). DECT acquisitions, VMI techniques, and reconstruction algorithms vary between manufacturers and types of DECT scanners. As a result, monochromatic energy levels cannot be reproduced between manufacturers or post-processing algorithms, thereby limiting study generalizability and indicating that different institutions may need to determine VMI optima independently [25].

In conclusion, use of virtual monochromatic images at 80–90 keV with non-contrast dual-energy CT results in a modest improvement of sensitivity and specificity for the detection of acute infarcts in suspected ischemic stroke patients relative to conventional non-contrast CT.

## Electronic supplementary material

ESM 1 (DOCX 15 kb)

## Abbreviations

AIS: Acute ischemic stroke
DECT: Dual-energy CT
VMI: Virtual monochromatic images
NCT: Conventional non-contrast head CT
mASPECTS: Modified ASPECTS
HU: Hounsfield units
WL: Window level
WW: Window width
PPV: Positive predictive value
NPV: Negative predictive value
IQR: Interquartile range
