# Supplementary material for: Virtual monochromatic dual-energy CT reconstructions improve detection of cerebral infarct in patients with suspicion of stroke
Source: Neuroradiology. 2020 Jul 29;63(1):41–9. doi: 10.1007/s00234-020-02492-y (PMC7803871; doi:10.1007/s00234-020-02492-y)
Supplement: Supplementary file 1 — (DOCX 15 kb) [file 234_2020_2492_MOESM1_ESM.docx]

Table 1: Ischemia detection as a function of time

|  | Sensitivity (R1,R2) | Specificity (R1,R2) | PPV (R1,R2) | NPV (R1,R2) | Accuracy (R1,R2) | IRR (95% CI) |
| --- | --- | --- | --- | --- | --- | --- |
| ≤4.5 hours | | | | | | |
| CCT | 18.0 (24.0,12.0) | 90.0 (96.0,84.0) | 64.3 (85.7,42.9) | 52.3 (55.8,48.8) | 54.0 (60.0,48.0) | 0.66 (0.49-0.84) |
| 60 keV | 12.0 (12.0,12.0) | 80.0 (92.0,68.0) | 43.6 (60.0,27.3) | 47.4 (51.1,43.6) | 46.0 (52.0,40.0) | 0.72 (0.55-0.88) |
| 70 keV | 26.0 (24.0,28.0) | 82.0 (88.0,76.0) | 60.3 (66.7,53.8) | 52.5 (53.7,51.4) | 54.0 (56.0,52.0) | 0.67 (0.48-0.85) |
| 80 keV | 26.0 (28.0,24.0) | 90.0 (96.0,84.0) | 73.8 (87.5,60.0) | 54.8 (57.1,52.5) | 58.0 (62.0,54.0) | 0.79 (0.65-0.93) |
| 90 keV | 22.0 (24.0,20.0) | 94.0 (100.0,88.0) | 81.3 (100.0,62.5) | 54.6 (56.8,52.4) | 58.0 (62.0,54.0) | 0.79 (0.65-0.94) |
| >4.5 hours | | | | | | |
| CCT | 46.2 (42.3,50.0) | 92.5 (100.0,85.0) | 90.6 (100.0,81.3) | 56.9 (57.1,56.7) | 66.3 (67.4,65.2) | 0.61 (0.41-0.81) |
| 60 keV | 48.1 (38.5,57.7) | 92.5 (100.0,85.0) | 91.7 (100.0,83.3) | 58.1 (55.6,60.7) | 67.4 (65.2,69.6) | 0.68 (0.49-0.86) |
| 70 keV | 46.2 (42.3,50.0) | 92.5 (100.0,85.0) | 90.6 (100.0,81.3) | 56.9 (57.1,56.7) | 66.3 (67.4,65.2) | 0.63 (0.42-0.83) |
| 80 keV | 46.2 (50.0,42.3) | 95.0 (100.0,90.0) | 91.7 (100.0,83.3) | 56.8 (60.6,52.9) | 66.3 (71.7,60.9) | 0.62 (0.42-0.83) |
| 90 keV | 50.0 (50.0,50.0) | 95.0 (100.0,90.0) | 93.3 (100.0,86.7) | 59.3 (60.6,58.1) | 69.6 (71.7,67.4) | 0.77 (0.61-0.94) |

Ischemia detection of VMI and conventional CT (CCT) in the early- (≤4.5 hours) and late-windows (>4.5 hours) after last-seen-well shown in sensitivity (%), specificity (%), PPV (%), NPV (%), accuracy (%) and inter-reader reliability (IRR), with 95% confidence interval (CI). The sensitivity, specificity, PPV, NPV and accuracy of each individual observer are provided parenthetically after the pooled value.
